# Medical Fees

**Published:** 1874-04

**Authors:** 


Medical Fees.—In Great Britain a decided attempt is making toward increasing the traditional consultation fee of a guinea to double that amount. It appears from the discussions on the subject in our English contemporaries, that the fee of one guinea was fixed in the latter half of the seventeenth century, in Charles II's reign, when money, estimated at its purchasing value in beef, bread and similar necessaries in life, was worth nearly thrice its present value. Hence, if the price put upon a physician's opinion in that day was correct, at this epoch two guineas is a very reasonable fee. A very elaborate comparison of the fluctuation of prices, and a close inquiry into professional social duties have been instituted, and it looks as if the change will be generally adopted.

In Canada a similar advance on the old fee-bills of thirty years ago, has already been adopted, and so far as we hear, without grumbling on the part of the public.

In this city, the College of Physicians and Surgeons have substantially abrogated the fee-bill altogether. It has proved next to impossible, if not wholly so, for any one to live up to it, and it is certainly better to dispense with a fee-bill which is neither legally valid nor practically respected.

The public are aware that they must pay more for the advice of one physician than for that of another; they expect it, and it is right that the hardest student, the most earnest worker, he who by years of labor has reached eminence in his profession, should place a higher price on his opinion than his lazy neighbor, who rarely ventures into profounder depths of science than his rusty text-books contain.

The objection that the profession should not be placed on a mere money basis, that it is derogatory to it to talk of it as a money-getting trade, is too affected, and savors too much of "buncombe" to be worth answering, hardly worth mentioning.

To speak of what is more important to our mind than the regulation of fees,—the collection of them; would that a general rule was adopted in city and country, wherever at all feasible, to present bills monthly, and urge monthly settlements. It is no uncommon matter for physicians to lose one-third or one-fourth of what is justly due them for services rendered, and much of this owed by parties who can pay, if they will. The general adoption of "black lists," registers, not of the worthy poor, but of the unworthy well-to-do, who shirk their doctor's bill, and change their attendant when he becomes pressing, ought to be generally kept and often consulted. An increased attention to this financial aspect of the profession would benefit the standing of physicians and the morals of their patients.

